# Quantifying lumbar mobility using a single tri-axial accelerometer

**DOI:** 10.1016/j.heliyon.2024.e32544

**Published:** 2024-06-06

**Authors:** David W. Evans, Ian T.Y. Wong, Hoi Kam Leung, Hanyun Yang, Bernard X.W. Liew

**Affiliations:** a School of Sport, Exercise and Rehabilitation Sciences, College of Life and Environmental Sciences, University of Birmingham, Birmingham, United Kingdom; b School of Sport, Rehabilitation and Exercise Sciences, University of Essex, Colchester, Essex, United Kingdom

**Keywords:** Lumbar mobility, Low back pain, Tri-axial accelerometer, Inertial measurement unit, Remote monitoring, Flexion velocity

## Abstract

**Background:**

Lumbar mobility is regarded as important for assessing and managing low back pain (LBP). Inertial Measurement Units (IMUs) are currently the most feasible technology for quantifying lumbar mobility in clinical and research settings. However, their gyroscopes are susceptible to drift errors, limiting their use for long-term remote monitoring.

**Research question:**

Can a single tri-axial accelerometer provide an accurate and feasible alternative to a multi-sensor IMU for quantifying lumbar flexion mobility and velocity?

**Methods:**

In this cross-sectional study, 18 healthy adults performed nine repetitions of full spinal flexion movements. Lumbar flexion mobility and velocity were quantified using a multi-sensor IMU and just the tri-axial accelerometer within the IMU. Correlations between the two methods were assessed for each percentile of the lumbar flexion movement cycle, and differences in measurements were modelled using a Generalised Additive Model (GAM).

**Results:**

Very high correlations (*r* > 0.90) in flexion angles and velocities were found between the two methods for most of the movement cycle. However, the accelerometer overestimated lumbar flexion angle at the start (-4.7° [95 % CI -7.6° to -1.8°]) and end (-4.8° [95 % CI -7.7° to -1.9°]) of movement cycles, but underestimated angles (maximal difference of 4.3° [95 % CI 1.4° to 7.2°]) between 7 % and 92 % of the movement cycle. For flexion velocity, the accelerometer underestimated at the start (16.6°/s [95%CI 16.0 to 17.2°/s]) and overestimated (-12.3°/s [95%CI -12.9 to -11.7°/s]) at the end of the movement, compared to the IMU.

**Significance:**

Despite the observed differences, the study suggests that a single tri-axial accelerometer could be a feasible tool for continuous remote monitoring of lumbar mobility and velocity. This finding has potential implications for the management of LBP, enabling more accessible and cost-effective monitoring of lumbar mobility in both clinical and research settings.

## Highlights

• Lumbar flexion angle and velocity were compared using an IMU and the accelerometer within.
• High correlations were found between the two methods for most of the movement.
• Greatest errors with the accelerometer occurred at the start and end of movement.
• Accelerometers have potential for continuous remote monitoring of lumbar mobility.

## 1 Introduction

Lumbar spine mobility is considered to be important for understanding the risk of low back pain (LBP) onset [[1], [2], [3]], its recovery [4], and its persistence [5,6]. Flexion is the lumbar spine movement most impaired in people with LBP [[7], [8], [9]]. Lumbar flexion is essential for many activities of daily living (ADLs), including lower limb dressing and picking objects up from the floor, which require 47 % and 73 % of maximal flexion-extension range of motion (ROM) respectively (Bible et al., 2010; Cobian et al., 2013). In addition to lumbar flexion mobility, flexion velocity has been identified as an important movement signature that characterises people with LBP compared to controls [10].

Gold-standard methods for measuring lumbar mobility are either video fluoroscopy [11] or tracking the motion of bone-pins [12]. Neither of these techniques can be adopted routinely in clinical settings or in population research studies. Lumbar mobility can be assessed using less harmful and invasive techniques, such as optical motion capture cameras, which track surface reflective markers placed onto the skin surrounding the lumbar region [13]. Significant limitations surround the use of cameras for assessing lumbar mobility, not least the significant financial cost of equipment, the lack of portability of the equipment, and the time required for data collection and processing. Accordingly, inertial measurement units (IMUs), which are small, portable, and relatively inexpensive, currently represent the most feasible technology for routinely quantifying lumbar mobility within clinics and population research studies [14,15].

IMUs are multi-sensor devices comprising tri-axial accelerometers, gyroscopes, and often magnetometers. One challenge when using IMUs to quantify lumbar mobility is that the technology is susceptible to drift errors due to low-frequency gyroscopic bias drifts, which are exacerbated with prolonged data collection [16]. This means that IMUs are excellent technologies for short-term use but are not suitable for long-term continuous remote monitoring in free-living environments for rehabilitation or research purposes. By contrast, when used in isolation, tri-axial accelerometers have the potential of being able to provide drift-free estimates of lumbar mobility whilst sensing the gravity vector [16,17]. A single tri-axial accelerometer has previously been used on the thigh to quantify not only thigh orientation, but also velocity during sit-to-stand movements in free-living environments [17].

To our knowledge, no study to date has measured lumbar flexion mobility and velocity using an IMU and compared these measurements to those captured using only a tri-axial accelerometer. If the measurements obtained using a single accelerometer are just as accurate as those obtained using a multi-sensor IMU, a single accelerometer would be considered a good candidate for continuous, long-term remote monitoring of lumbar mobility and velocity. The objective of this study was to compare these two approaches.

## 2 Methods

### 2.1 Hypothesis

Our hypothesis is that the angle and velocity of lumbar flexion, measured by a single tri-axial accelerometer in isolation, will have a high correlation with equivalent values measured from a multi-sensor IMU.

### 2.2 Study design and participants

The study was cross-sectional by design, with recruitment and data collection taking place between November 2022 to February 2023 at the University of Essex, UK. Healthy participants were eligible to participate if they met the following inclusion criteria: 1) aged between 18 and 40 years, 2) free from any known restrictions in lumbar spinal mobility, and 3) currently free from symptoms emanating from the lumbar spine region, as self-reported. The study design was approved by the University of Essex Ethics Ethics Committee (ETH2223-0474). All participants provided electronic signed informed consent prior to study participation.

### 2.3 Sample size

In order to detect a moderate correlation of 0.6 between angles measured using the two methods, at a power of 0.8 and a Bonferroni corrected alpha of 0.05, 16 participants were required. To allow for data loss, we sought to recruit 18 participants.

### 2.4 Instruments

**Fig. 1:**
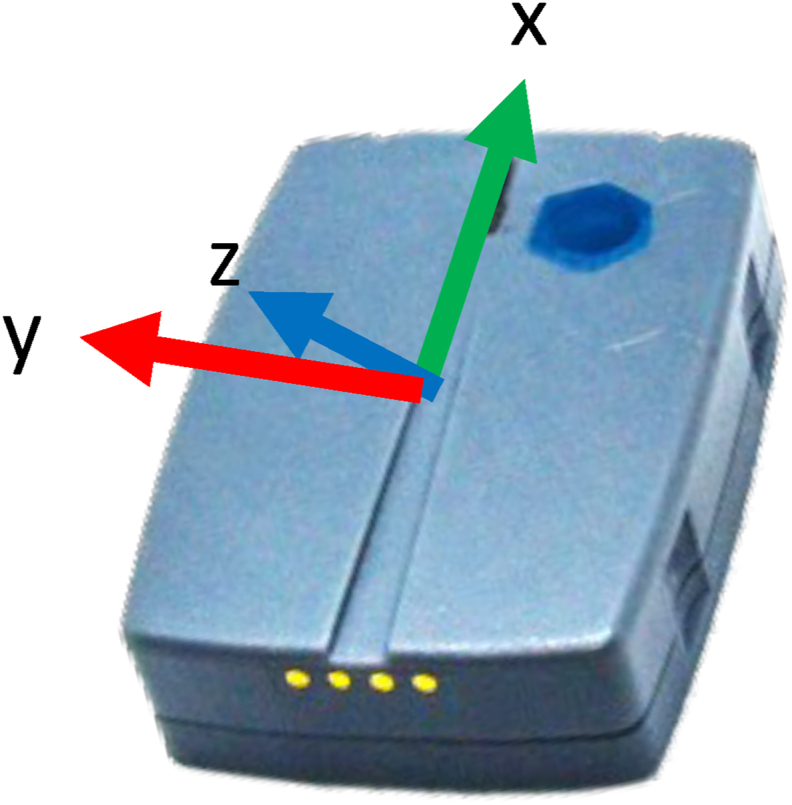
Axis of the inertial measurement unit, x - pointing upward, y - pointing left, and z point outward (towards reader), when using the right-hand rule.

A laboratory-standard IMU (Noraxon, USA) with a 200Hz sampling rate, which incorporated a tri-axial accelerometer, gyroscope and magnetometer, was positioned on the skin overlying the spinous process of the third lumbar (L3) vertebra, which was located via manual palpation and anatomical landmarks [18]. The IMU was secured using the manufacturer's elastic Velcro straps (Fig. 1). A Toshiba Portege laptop was used to run the manufacturer's Noraxon MyoResearch software (MR3 3.18.98) and capture all sensor data, including raw accelerometry signals and combined and processed multi-sensor data.

### 2.5 Procedures

Age (years), gender, body mass (kg), and height (m) were collected via self-report. For all procedures, participants were instructed to stand with their feet shoulder-width apart, and pointing forwards, as judged by the assessor. Participants performed three sets of three consecutive repetitions of full spinal flexion movements (i.e., nine repetitions in total). A static rest period of 1 min duration in the standing position was used between sets. Using metronome software (Pro Metronome, free version) on a tablet computer (iPad, Apple Computers, Cupertino, CA) set to 20 beats per minute [19], all spinal movements were performed at a fixed pace with each half-cycle of movement matched to the metronome frequency (e.g. ‘beat’ neutral, ‘beat’ maximally flexed, ‘beat’ neutral, etc.). The IMU sensor was calibrated prior to the start of each set with participants stood in an upright position. Participants were given an opportunity to familiarise themselves with the task prior to data collection, to ensure synchronisation of movements with the metronome cadence. Participants were blinded to all data collected.

### 2.6 Signal processing

Flexion angles were calculated using the multi-sensor IMU and separately using just the tri-axial accelerometer within the same IMU device. Two events were visually identified from the lumbar segment flexion angle signal, for each flexion repetition: ‘start’ where the flexion manoeuvre began, and ‘end’ where the participant returned to the upright position. Five signals recorded between these events were exported from the Noraxon data for further processing: three-dimensional (3D) linear acceleration signals from the accelerometer, lumbar segment flexion angle from the IMU (${Flex}_{IMU}$), and lumbar segment gyroscope velocity from the IMU (${Vel}_{IMU}$). Lumbar segment flexion angle (in degrees) was derived from the 3D linear acceleration signals (${Flex}_{acc}$), using Equation (1) [17], and lumbar segmental flexion velocity (${Vel}_{acc}$) was derived by taking the first derivative of ${Flex}_{acc}$.

(1) $${\text{Flex}}_{\text{acc}}\left({}^{\circ}\right)=90‐\tan^{‐1}\frac{{\text{acc}}_{x}}{\sqrt{{{\text{acc}}_{y}}^{2}+{{\text{acc}}_{z}}^{2}}}\times \frac{180}{\pi }$$

Where ${acc}_{x}$, ${acc}_{y}$, ${acc}_{z}$ were the linear accelerations along the x, y, and z axis, respectively. The signals were segmented between the two ‘start’ and ‘end’ events, and time-normalised to 100 data points. The four signals, two flexion angles and two velocities, were filtered with a 4th order, zero-lag Butterworth filter at 1Hz to reduce ‘noise’ caused by skin motion artefact [20].

### 2.7 Statistical inference

All statistical analyses were performed using R (version 4.2.2). Data were analysed by a member of the research team (BXWL) not directly involved in data collection. We performed a multilevel correlation [21] between the two methods for deriving flexion angle and velocity at each 1 % of the movement cycle [22]. The following thresholds were used for interpretation of the magnitude of correlation coefficient: |0to0.30| represents negligible correlation, |>0.30to0.50| reflects low correlation, |>0.50to0.70| reflects moderate correlation, |>0.70to0.90| reflects high correlation, and |>0.90| reflects very high correlation [23].

We quantified error as the difference in measurements of angles and velocity between the two methods (IMU measurement minus that of accelerometer). Next, we modelled the dependent variables of Δangle and Δvelocity of subject *i*, at the *j*th % cycle point as a Generalised Additive Model (GAM) [24], given by Equations (2), (3)) respectively.

(2) $${\Delta angle}_{ij}=\alpha +\beta_{subject,i}+f\left({cycle}_{j}\right)+\varepsilon_{ij}$$

(3) $${\Delta velocity}_{ij}=\alpha +\beta_{subject,i}+f\left({cycle}_{j}\right)+\varepsilon_{ij}$$

where the dependent variables are modelled by a global intercept *α*, a random intercept $\beta_{subject,i}$ for every subject, and a smooth cycle effect $f\left({cycle}_{j}\right)$ [24]. This statistical approach enables us to treat the entire angular or velocity waveform of a single repetition across the movement cycle as a single observation. This eliminates the need for repeated statistical tests at each time point. The amount of penalization controlled by the smoothing parameter of the quadratic penalty determining the ‘wiggliness’ of the non-linear effects of cycle time was estimated using a generalised cross-validation approach [25]. Statistical significance was defined when the 95 % CI of the smoothed effects did not contain zero [26].

## 3 Results

Eighteen adult participants (9 males, 9 females; mean [standard deviation] age 20.8 [1.6] years, body mass 65.8 [12.4] kg, and height 1.7 [0.09] m) who met the eligibility criteria were recruited. All participants provided data.

**Fig. 2:**
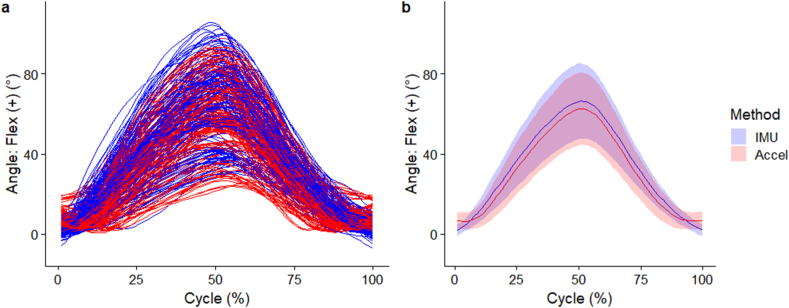
(a) Individual waveforms, (b) group wise mean (standard deviation as error clouds) of lumbar segment flexion angle. Blue – angles from IMU directly, Red – angles from accelerometers. (For interpretation of the references to colour in this figure legend, the reader is referred to the Web version of this article.)

**Fig. 3:**
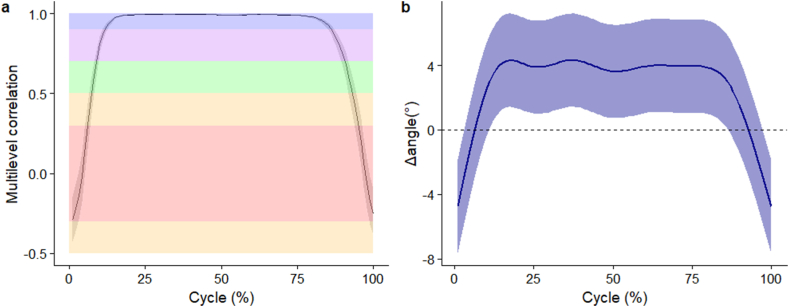
(a) Multilevel correlation (95 % confidence interval as error clouds) between angles, (b) mean difference (95 % confidence interval as error clouds) of lumbar segment flexion angles. Horizontal shaded areas reflect correlation thresholds of red (negligible), orange (very low), green (moderate), purple (high), and blue (very high). (For interpretation of the references to colour in this figure legend, the reader is referred to the Web version of this article.)

The individual and mean (SD) waveforms of lumbar segment flexion angle are seen in Fig. 2a and b respectively. The flexion angles elicited by the two methods were very highly correlated (*r* > 0.90) between 12 % and 86 % of the movement cycle but had negligible correlations near the start and end of the movement cycles (Fig. 3a). Compared to the IMU method, the accelerometer method overestimated lumbar segment flexion angle at the start (-4.7° [95 % CI -7.6° to -1.8°]) and end (-4.8° [95 % CI -7.7° to -1.9°]) of movement cycles (Fig. 3b). The accelerometer method underestimated lumbar segment flexion angle between 7 % and 92 % of the movement cycle. Peak underestimation using the accelerometer method occurred at 37 % cycle, with a difference of (4.3° [95 % CI 1.4 to 7.2°]) (Fig. 3b).

**Fig. 4:**
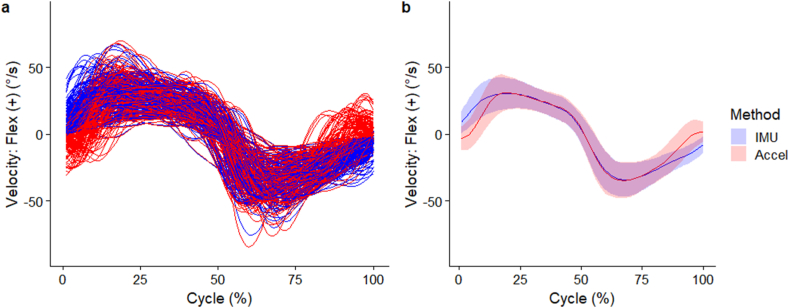
(a) Individual waveforms, (b) group wise mean (standard deviation as error clouds) of lumbar segment flexion velocities. Blue – angles from IMU directly, Red – angles from accelerometers. (For interpretation of the references to colour in this figure legend, the reader is referred to the Web version of this article.)

**Fig. 5:**
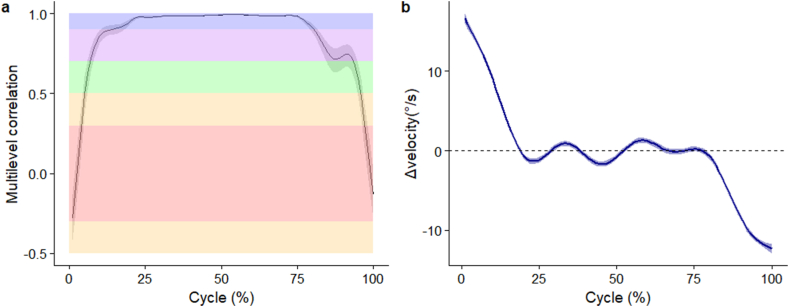
(a) Multilevel correlation (95 % confidence interval as error clouds) between angles, (b) mean difference (95 % confidence interval as error clouds) of lumbar segment flexion velocities. Horizontal shaded areas reflect correlation thresholds of red (negligible), orange (very low), green (moderate), purple (high), and blue (very high). (For interpretation of the references to colour in this figure legend, the reader is referred to the Web version of this article.)

The individual and mean (SD) waveforms of lumbar segment flexion angle are seen in Fig. 4a and b respectively. Maximal flexion velocity occurred at 20 % cycle with a magnitude of 30.3 (11.6)°/s. Maximal extension velocity occurred at 68 % cycle with a magnitude of -34.5 (12.6)°/s. Very high correlations (*r* > 0.90) existed between the two methods to elicit flexion velocities between 14 % and 80 % of the movement cycle but correlations were negligible near the start and end of the movement cycles (Fig. 5a). The accelerometer method underestimated lumbar segment flexion velocity at the start (16.6°/s [95%CI 16.0 to 17.2°/s]), and overestimated velocity at the end (-12.3°/s [95%CI -12.9 to -11.7°/s]) of movement, compared to the IMU method (Fig. 5b). Between 14 % and 85 %, the absolute difference in velocities was <5°/s, whilst between 18 % and 80 %, the absolute difference was <1°/s (Fig. 5b).

## 4 Discussion

This study compared lumbar segment flexion and extension angles that were calculated using a lumbar-worn multi-sensor IMU and using just the tri-axial accelerometer within the same IMU device. The findings from this study partially confirm our hypothesis that the lumbar flexion angle and velocity, calculated from just a tri-axial accelerometer, will have a high correlation with equivalent values measured from a multi-sensor IMU. However, this hypothesis must be rejected for motion occurring at the beginning and end of the movement cycle. At these extremes, the accelerometer method overestimated lumbar segment flexion angle by ∼5°. Likewise, the accelerometer method underestimated lumbar segment flexion velocity at the start by ∼17°/s, and overestimated velocity at the end ∼13°/s of movement, compared to the IMU method. By contrast, the accelerometer method underestimated lumbar segment flexion angle between 7 % and 92 % of the movement cycle.

Care is needed when interpreting the angles and velocities provided by the accelerometer method. The largest difference observed was on average <5°, with a maximal magnitude of difference of ∼8°. By comparison, using an electromagnetic tracking device, Shum and colleagues [27] reported a difference of 23° between individuals with and without LBP, in maximal lumbar flexion when putting on a sock. In addition, a case-series by Wernli et al. (2020) [28] documented a substantial reduction in LBP intensity alongside an approximate 25° modification in the trunk segmental flexion angle. However, it is worth noting that they observed day-to-day fluctuations in mobility to be less than 5°.

For velocity, the observed differences between the methods (13°/s compared to 17°/s) are likely to be clinically important. Differences in lumbar flexion velocity between people with and without LBP have been reported to be 17.8°/s during a pick-up task, 16.5°/s during a lifting task, and 8.1°/s during a pure standing flexion task [10]. Based on these findings, it can be inferred that utilizing the accelerometer method may not be suitable for accurately measuring minor fluctuations in lumbar mobility but may still provide useful information regarding lumbar angles during daily activities.

The differences in angle and velocity between using an accelerometer-only and the IMU are consistent with the literature. When comparing the error in angles obtained from an accelerometer placed on a mechanical device, previous studies reported an error of <1.5° [29,30]. One study reported an upper trunk (T1 spinal level) root mean squared error (RMSE) ranging between 2.5° and 5.4°, when comparing an accelerometer against the reference of a magnetic tracking device [31]. Previous studies have reported that accelerometers alone can measure angles and velocities accurately at low movement speeds, but not at faster speeds. For example, when comparing to flexion angles obtained by optical motion cameras, the RMSE for angles increased from 2.3° when moving at 15 cycles/min to 11.3° at 45 cycles/min [32]. The RMSE for velocity increased from 13°/s at 15 cycles/min to 79°/s at 45 cycles/min [32]. An increase in error with greater movement speeds may be associated with increases in tangential and centripetal acceleration with greater movement speeds [33].

Lumbar flexion-extension is arguably one of the most common movements performed in daily living and one of the most affected by the presence of LBP. Accelerometers are already being used to study physical activity levels in LBP [34]. The present study has demonstrated that if the accelerometer is placed on the low back for physical activity monitoring [34], it can simultaneously be used for tracking lumbar mobility alterations in daily living scenarios. This may provide a better objective marker of functional recovery in LBP, thereby serving as a better endpoint outcome within clinical trials and in clinical practice. The clinical importance of a greater increase in errors in the measured angle and velocity using an accelerometer alone, may be different depending on the intended utility of wearable sensors to measure lumbar mobility variables. For example, the use of inclinometry when evaluating occupational risk hazards largely requires the assessment of time spent in several categories of postures [35]. Some categories may include the time spent in a lumbar flexion posture of <30° vs ≥60° [32]. A tendency for greater errors in angle estimation with higher movement speeds will likely not hinder estimation of time spent in specific postures or positions.

This study is not without limitations. Firstly, we investigated the differences between sensors at one movement speed using a highly controlled sagittal plane movement (lumbar flexion). The accuracy of the accelerometer method may be affected during functional movement tasks, such as lifting, where movement speeds may vary within and between motion cycles, and that movement will likely occur across all three planes. Secondly, we tested the accuracy of a tri-axial accelerometer against a research grade IMU, and not a commercially available product. Although potentially reducing the generalisability of the present study, this enabled us to ensure precise temporal synchronisation between angles derived from the two sources – an important procedure for the validation of the entire waveform.

## 5 Conclusions

An accelerometer attached to the lumbar spine will provide lumbar flexion angles that are highly correlated with those measured by a multi-sensor IMU at the same location for all but the first and last 20 % of the standing bending (lumbar flexion) movement cycle. Angles measured by the two methods are likely to be within 8° degrees of each other throughout the movement cycle. The angles recorded by the accelerometer are likely to be slightly overestimated at the start and end (first and last 8 %) of the cycle and slightly underestimated for the remainder of the cycle. This result will be useful to inform remote clinical monitoring of lumbar motion during rehabilitation and population research.

## CRediT authorship contribution statement

**David W. Evans:** Writing – review & editing, Methodology, Conceptualization. **Ian T.Y. Wong:** Writing – review & editing, Project administration, Investigation, Data curation. **Hoi Kam Leung:** Writing – review & editing, Project administration, Investigation, Data curation. **Hanyun Yang:** Data curation, Investigation, Project administration, Writing – review & editing. **Bernard X.W. Liew:** Writing – review & editing, Writing – original draft, Visualization, Validation, Supervision, Software, Project administration, Methodology, Investigation, Formal analysis, Data curation, Conceptualization.

## Declaration of competing interest

The authors declare that they have no known competing financial interests or personal relationships that could have appeared to influence the work reported in this paper.
